# Association between air pollution from residential wood burning and dementia incidence in a longitudinal study in Northern Sweden

**DOI:** 10.1371/journal.pone.0198283

**Published:** 2018-06-13

**Authors:** Anna Oudin, David Segersson, Rolf Adolfsson, Bertil Forsberg

**Affiliations:** 1 Occupational and Environmental Medicine, Department of Public Health and Clinical Medicine, Umeå University, Umeå, Sweden; 2 Swedish Meteorological and Hydrological Institute, Norrköping, Sweden; 3 Division of Psychiatry, Department of Clinical Sciences, Umeå University, Umeå, Sweden; Telethon Institute for Child Health Research, AUSTRALIA

## Abstract

**Objectives:**

There is highly suggestive evidence for an effect of air pollution exposure on dementia-related outcomes, but evidence is not yet present to clearly pinpoint which pollutants are the probable causal agents. The aims of this study was to assess the longitudinal association between exposures of fine ambient particulate matter (PM_2.5_) from residential wood burning, and vehicle exhaust, with dementia.

**Method:**

We used data from the Betula study, a longitudinal study of dementia in Umeå, Northern Sweden. The study size was 1 806 and the participants were followed from study entry (1993–1995) to 2010. Modelled levels of source-specific fine particulate matter at the residential address were combined with information on wood stoves or wood boilers, and with validated data on dementia diagnosis and individual-level characteristics from the Betula study. Cox proportional hazards models were used to estimate Hazard Ratios (HRs) and their 95% CIs for dementia incidence (vascular dementia and Alzheimer’s disease), adjusted for individual-level characteristics.

**Results:**

The emission of PM_2.5_ from local residential wood burning was associated with dementia incidence with a hazard ratio of 1.55 for a 1 μg/m^3^ increase in PM_2.5_ (95% Confidence Interval (CI): 1.00–2.41, p-value 0.05). Study participants with an address in an area with the highest quartile of PM_2.5_ from residential wood burning and who also had a wood-burning stove were more likely to develop dementia than those in the lower three quartiles without a wood-burning stove with hazard ratios of 1.74 (CI: 1.10–2.75, p-value 0.018). Particulate matter from traffic exhaust seemed to be associated with dementia incidence with hazard ratios of 1.66, (CI: 1.16–2.39), p-value 0.006, and 1.41 (CI: 0.97–2.23), p-value 0.07, in the third and fourth quartiles, respectively.

**Conclusions:**

If the associations we observed are causal, then air pollution from residential wood burning, and air pollution from traffic, might be independent important risk factors for dementia.

## Introduction

Dementia is a major cause of morbidity and mortality worldwide. By 2050, the number of Alzheimer’s Disease (AD) patients is expected to rise to over 130 million from today’s 47 million [1]. The mechanisms behind dementia are not fully understood but likely includes genetic, physiological and cognitive factors.

In a study from Northern Sweden, we have previously observed residential concentrations of traffic-related air pollution to be a risk factor for vascular dementia and AD, with an attributable fraction of 16% [2]. Chen and colleagues later showed that living close to heavy traffic was associated with dementia in Canada, with an adjusted hazard ratio (HR) of 1.07 (CI: 1.06–1.08) for people living less than 50 m from a major traffic road as compared with living further than 300 m[3]. In two early studies from Taiwan, NO_2_ was correlated with elevated risk of dementia, with a HR of 1.54 (CI: 1.34–1.77) in quartile 4 versus quartile 1[4], and a 4.34 μg/m^3^ increase in particulate matter 2,5 μm or less in diameter (PM_2.5_) was associated with an HR of 2.38 (CI: 2.21–2.56) [5]. Furthermore, Cacciottolo and colleagues found that residing in places with PM_2.5_ exceeding US Environmental Protection Agency standards increased the risks for dementia by 92% [6]. A review from 2016 concludes that it is highly plausible, that air pollution has an effect on cognition, but current evidences does not clearly pinpoint which pollutants are the probable causal agents[7].

Wood smoke is a natural substance why there is a general sentiment that it must be benign. However, it is scientifically well established that biomass burning emits health-damaging pollutants, including several carcinogenic compounds [8]. Domestic wood smoke at high levels, as when cooking is done indoors without chimneys[8], can cause hypertension, affect respiratory health[9] and artery intima-media thickness, cause a rise in the prevalence of atherosclerotic plaques[10] and even cause lung cancer [11]. Experimental evidence indicate that particles stemming from wood burning may have similar health effects as particles stemming from traffic [8, 12, 13]. Indoor wood smoke at high doses is thus a well-established cause of morbidity and mortality, but little is known about the health effects at lower doses. Small-scale heating may be a substantial contributor to ambient PM_2.5_ levels in certain residential areas, in high-income countries. For example, the biomass contribution to air pollution levels in small country towns in Sweden can be extensive, leading to levels of particles comparable to busy street canyons in major cities [12]. Due to climate mitigation policies, there are incentives leading to an increase in biomass burning, but knowledge of the health effects of such policy is scant [14, 15].

It has not been possible in previous epidemiological studies to investigate air pollution from residential wood burning as a possible causal agent because to do so it is necessary to resolve the strong gradients close to major emission sources, such as appliances for small scale residential heating. Such detailed exposure data is now available in parts of Sweden. With that exposure data, PM from wood burning was estimated to cause a larger or comparable number of annual deaths (depending on location and risk estimate used) than traffic-related air pollution [16]. Combined with data from a longitudinal cohort study on dementia, this detailed exposure data allow us to investigate our objectives; if exposure to particles from residential wood burning, and particles from vehicle exhaust, are associated with dementia incidence. In addition we use the information from chimney sweepers to study the effect of indoor sources.

## Methods

To determine whether there is a link between exposure to combustion particles and dementia incidence, we linked new source-specific particle data on fine particulate matter (PM_2.5_) with data from a previous study on traffic-related air pollution (where air pollution exposure was assessed by NO_x_ concentrations from a land use regression model) and dementia incidence [2].

Three data sets was used in this study. The first data set was longitudinal data from the Betula study on dementia diagnosis and individual-level characteristics which we used in our previous study[2]. The second and third data set was on emission sources (e.g. traffic flows and stoves) which yielded modelled levels of particle pollution and information on residential addresses from Statistics Sweden. The data sets were combined by linking the residential address at start of follow-up period in the Betula study with source-specific fine particulate air pollution levels from the 50 m x 50 m grid square in which each participant’s home was located.

### The betula study

**Fig 1:**
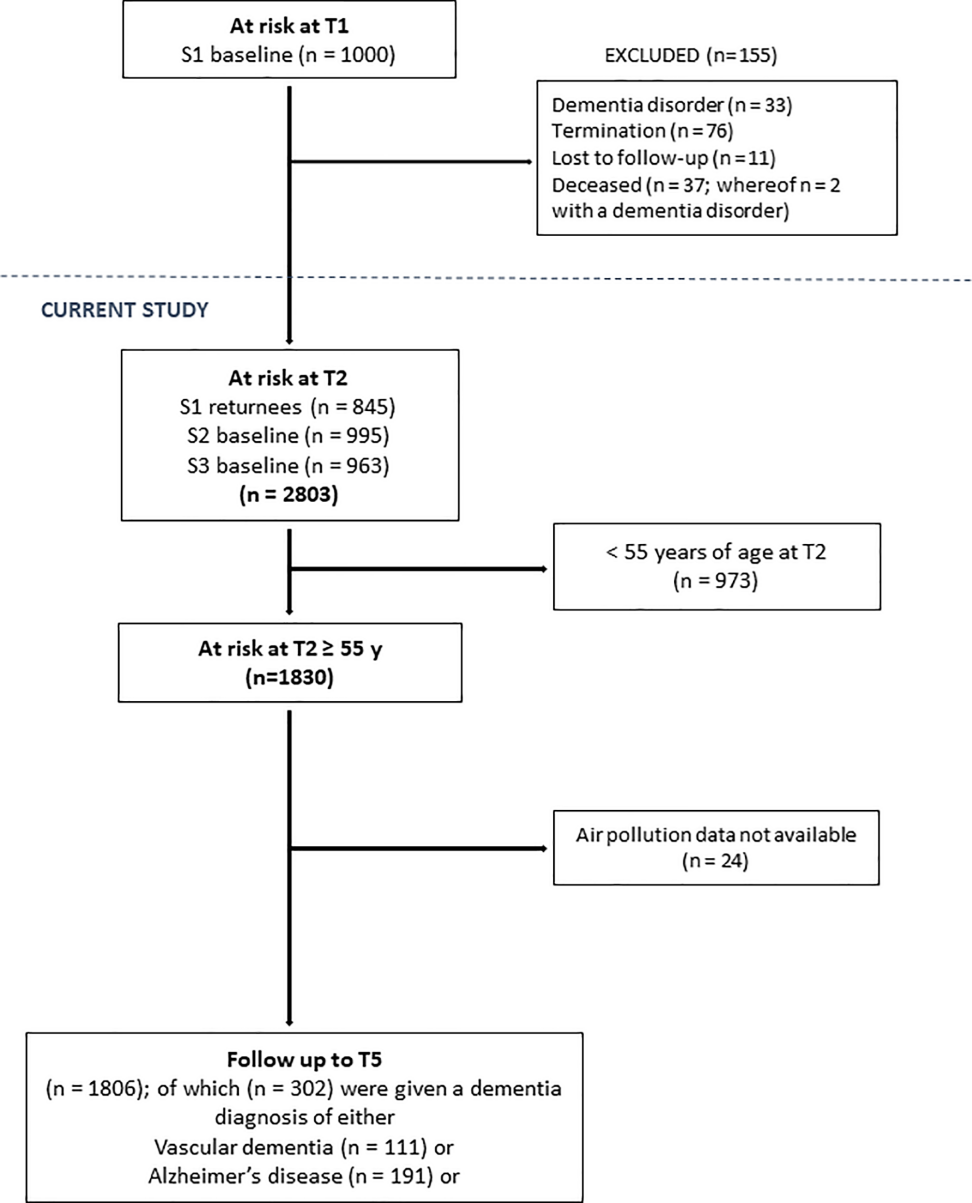
Flow-chart from study inclusion to end of follow-up.

The Betula study is a longitudinal cohort that was initially motivated by the need to explore various aspects of health and cognitive aging and dementia [17]. The Betula data used for the present study is described more in detail elsewhere [2]. At the first data collection in 1988–1990 (T1), an equal number of men and women, distributed among 10 age cohorts (all 35, 40, 45, … 80 years of age), were randomly sampled from the general population in the municipality of Umeå for a final sample of 1,000 participants. At the first follow-up, in 1993–1995 (T2), two new cohorts were included: sample 2 (S2; n = 995), with the same age and sex distribution as S1 at T1, and sample 3 (S3; n = 963), with the same age and sex distribution as S1 at the time of T2 (all 40, 45, 50, … 85 years). To date, data collection has been conducted six times with 5-year intervals between the follow-ups (T1, 1988–1990; T2, 1993–1995; T3, 1998–2000; T4, 2003–2005; T5, 2008–2010; T6, 2013–2014). At each test point (T1–T6), the investigation was split into two occasions; a health questionnaire was administered on the first occasion and a cognitive evaluation was made on the second. Between the first and second testing, study participants filled out a battery of self-assessment forms covering various socioeconomic, health, aging, and personality traits.[17] In the event of pathological findings, the study physician (Rolf Adolfsson, RA) was consulted, and if deemed necessary, the participant was referred to his/her primary health care practitioner for follow-up. The cognitive test battery included tasks to assess episodic memory, semantic memory, working memory, the perceptual representation system, prospective memory, visual attention, processing speed, problem solving, and decision-making. The present study starts at T2, why data on participants gathered at T2 were considered baseline information (Fig 1). The start of follow-up was thus 1993–1995 and the end of follow-up was 30^th^ of June 2010.

### Dementia diagnoses

In the Betula study, dementia status was assessed at baseline and reassessed every 5 years to identify new cases and to determine the year in which the DSM-IV (Diagnostic and Statistical Manual of Mental Disorders, 4th Edition) core criteria for dementia were met, that is, when cognitive symptoms became sufficiently severe to interfere with social functioning and with instrumental activities of daily living (American Psychiatric Association 2000). The medical records from all hospital and primary care visits within the county were continuously evaluated over the entire study period. The diagnoses were based on observations obtained at the Betula study visits (health and cognitive evaluations) supplemented with medical record data. Available clinical results from magnetic resonance imaging, computerized tomography scans, and autopsy (not part of the Betula protocol) were also considered in the diagnostic decision. The Betula study population (n = 4,445) was evaluated in this manner with regard to dementia after each test wave (T1, T2, T3, T4, T5). Evaluation of T6 has not been completed at the time of writing. The diagnostic evaluations were coordinated by the same senior research geropsychiatrist (RA) throughout the study period.

In 2011, an extensive quality assurance assessment of the dementia diagnoses in the Betula study was performed. In this assessment, a blinded re-evaluation was made of the medical records of those with an established dementia diagnosis with regard to dementia status, subtype, and age at onset [18]. 444 individuals with a dementia diagnosis were reevaluated by blinded investigators. The reevaluated diagnoses were based on a minimum of 5 years additional information about the course of the disease. As part of the Betula protocol, the following predetermined criteria, which were recorded at the health and cognitive examinations, were used as a guide for an extended evaluation as determined by the senior geropsychiatrist (RA): Mini Mental State Examination (MMSE) score ≤ 23, a decline in cognitive performance compared with a previous test occasion (from high to average/low or from average to low), a decline in daily functional activities or a subjective loss of memory function expressed by the participant in the semi-structured interview and any other behavioral or cognitive deviations such as confusion or disorientation noticed by the testing team.

Of the 1,806 participants included in the present study, 302 were diagnosed with either AD (n = 191) or vascular dementia (n = 111) before or during T5. In this context, it should be noted that a dementia diagnosis given to a participant from the S1 or S3 cohorts was based on both clinical examinations and medical record data obtained between 1993 and 2011, whereas for the S2 participants, a dementia diagnosis given in T4 was based solely on information obtained from medical records because these participants were not followed up after that point.

### Exposure modelling

To obtain estimates of exposure to source-specific PM_2.5_, we used data on the annual mean concentration of PM_2.5_ for 1990, 2000 and 2010, calculated by the Swedish Meteorological and Hydrological Institute (SMHI), described in detail elsewhere[16]. The concentrations were estimated using a wind model and a Gaussian air quality dispersion model. Road networks were described with a high level of detail, and measured traffic flow for heavy and light vehicles was collected separately by SMHI for most major roads and elsewhere was completed using SMHI’s modelled traffic flow. The vehicle fleet composition was derived from the National Vehicle Registry. Vehicles were grouped into passenger cars (petrol, diesel, ethanol, gas), light commercial vehicles (petrol and diesel), heavy goods vehicles (petrol and diesel) and buses (diesel, bio gas, and ethanol). Emission factors for exhaust for different vehicle types, speeds and driving conditions were calculated based on the Handbook Emission Factors for Road Transport (HBEFA) 3,1[19]. To obtain estimates of PM_2.5_ from residential heating, SMHI used a detailed emission inventory according to chimney-sweepers was collected by the Department for Occupational and Environmental Medicine, Umeå University allowing emissions to be represented as point sources. A total of 10,287 appliances (2006–2009) were represented as point sources within the assessment area. Emission factors are based on measurements reported by Todorovic [20], but adjusted following discussions by Nussbaumer [21], regarding different measurement technologies. The inventory was validated through a monitoring campaign in and around Umeå, with focus on areas with significant impact from small scale residential heating. In addition, a survey regarding wood consumption and firing habits was carried out, allowing estimation of the average wood consumption for different heating appliances. Further details regarding emission factors, validation of the inventory and the survey is presented by Omstedt et al. [22]. In our study area, the local particle emissions from residential heating were totally dominated by the wood burning, [16] why this particle source hereafter is labelled as “residential wood burning”. The model grids had an original spatial resolution of 3200x3200 m, but as the areas became more urban the resolution was successively improved to 50 m x 50 m. The main local contributors to combustion PM_2.5_ emissions were road traffic (vehicle exhaust) and residential wood burning.

The categories of stoves or boilers obtained from the chimney sweepers were: “wood stoves” (“open fireplace” or “cottage” were added), “wood boilers often used”, “wood boilers less often used”, “wood pellet boilers”, and “oil boilers”, “open fireplace” and “cottage”. The pellet and oil boilers were very rare, so we merged those categories in the analysis. Wood boilers are large appliances connected to the heating system of the house. Woods stoves are mostly used for pleasure and heating of smaller segments of the house. We merged “open fireplace” and “cottage” into the category “wood stoves”. We then linked the concentrations provided by SMHI to the geocoded home address of the Betula study participants as of December each year in 1990, 2000 and 2010 according to the Population Register. We also linked the home address of the study participants to information from the register of stoves and boilers to obtain information on individual access to stoves or boilers.

### Statistical analysis

Cox proportional hazards models were used with time as the underlying scale to calculate hazard ratios (HRs) and 95% confidence intervals (CIs) for dementia incidence in association with annual mean concentrations of PM_2.5_ from residential wood burning, traffic exhaust, and with type of stove or boiler. The mean follow-up time was 11.4 years. The two PM_2.5_ variables (vehicle exhaust and residential wood burning) were included in the statistical models simultaneously, both as continuous and categorical (quartiles) variables. We also created a new exposure variable by combining PM_2.5_ from residential wood burning with the presence of a wood stove in the home. Modelled annual PM_2.5_ levels at the residential address at study entrance were used as a proxy for long-term exposure to air pollution.

To reduce potential bias from residual confounding PM_2.5_ estimates were adjusted for the same set of potential confounding variables we used in our previous study [2]. These were: the categorical variables education level (low, medium, high), physical activity (low versus medium plus high), smoking (current versus former or non-smoker), sex (male versus female), body mass index (overweight versus non-overweight), waist-hip ratio (>recommended versus ≤recommended), alcohol (yes, no never, no quit), and age (55, 60, 65, 70, 75, 80, 85–95). Due to limited statistical power, we chose not to do any subgroup-analyses or interaction-analyses except that we stratified the analysis on sex. For more reasoning behind our choices of potential confounders, see our previous study [2]. We analyzed complete-case data. In an additional analysis, we adjusted the estimates for type of stove or boiler “none”, “wood stove”, “wood boiler often used”, “wood boilers less often used”, “pellet or oil”).

All participants in the Betula study gave written informed consent, and the study was approved by the Regional Ethics Review Board at Umeå University with DNR: 2012-12-31M.

## Results

Participants in S1 who had died, had received a dementia diagnosis, were lost to follow-up, or had left the study for other reasons before T2 were excluded (n = 155). At baseline (T2), the three samples consisted of 2,803 individuals without dementia; S1 (n = 845), S2 (n = 995), and S3 (n = 963). Participants less than 55 years of age at T2 (n = 973) were thereafter excluded because of their low risk of developing dementia within the coming 15-year follow-up period. Participants with addresses that could not be geocoded (n = 24) were also excluded from further analysis. Thus, the final sample consisted of 1,806 individuals (Fig 1).

**Fig 2:**
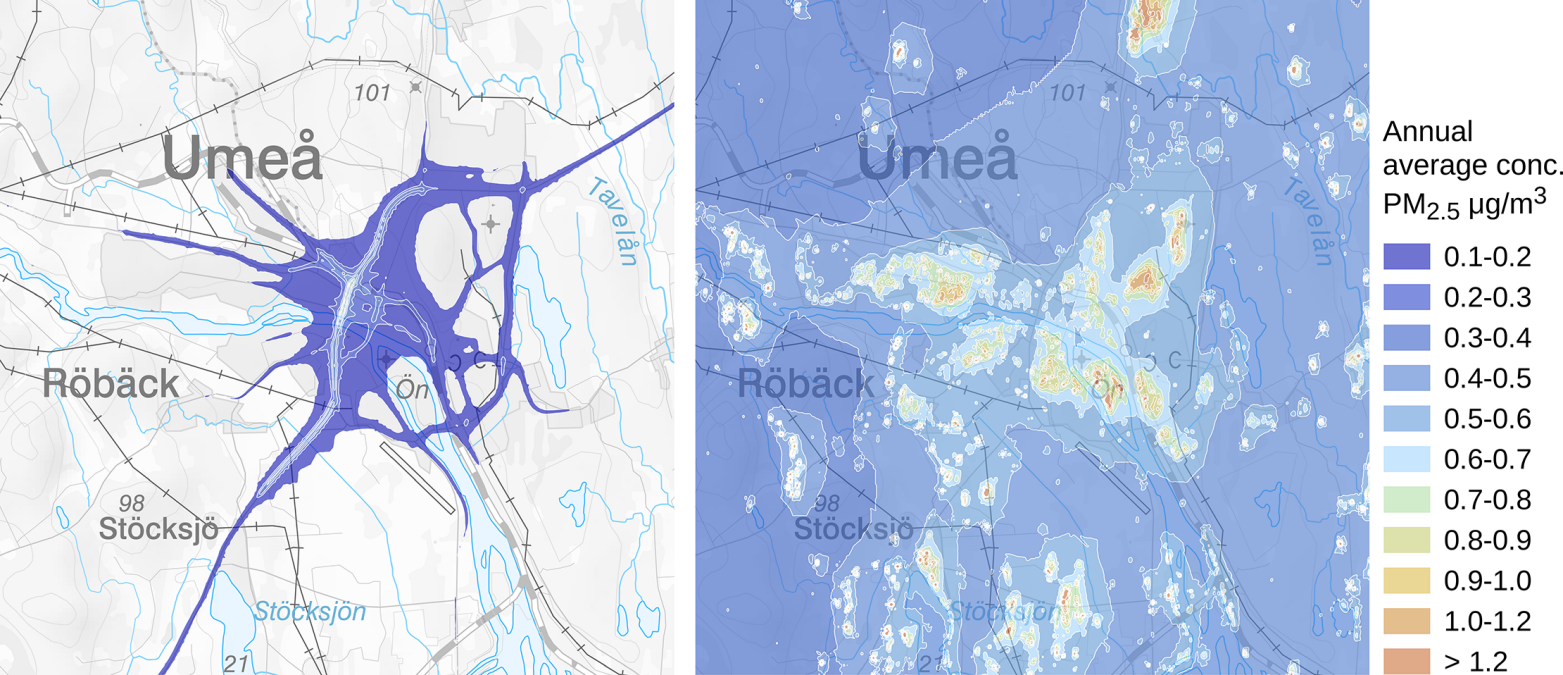
Modelled annual average contribution to PM_2.5_ during 2011 from road traffic exhaust (left map) and small scale residential wood burning (right map) by SMHI. Copyright Lantmäteriet.

The local contributions to PM_2.5_ of residential wood burning and vehicle exhaust in the study area are shown in Fig 2, with clear differences in gradients between the two sources (modelled annual average contribution to PM_2.5_ during 2011 from road traffic exhaust to the left and small scale residential heating, mostly wood-burning to the right; Fig 2).

**Fig 3:**
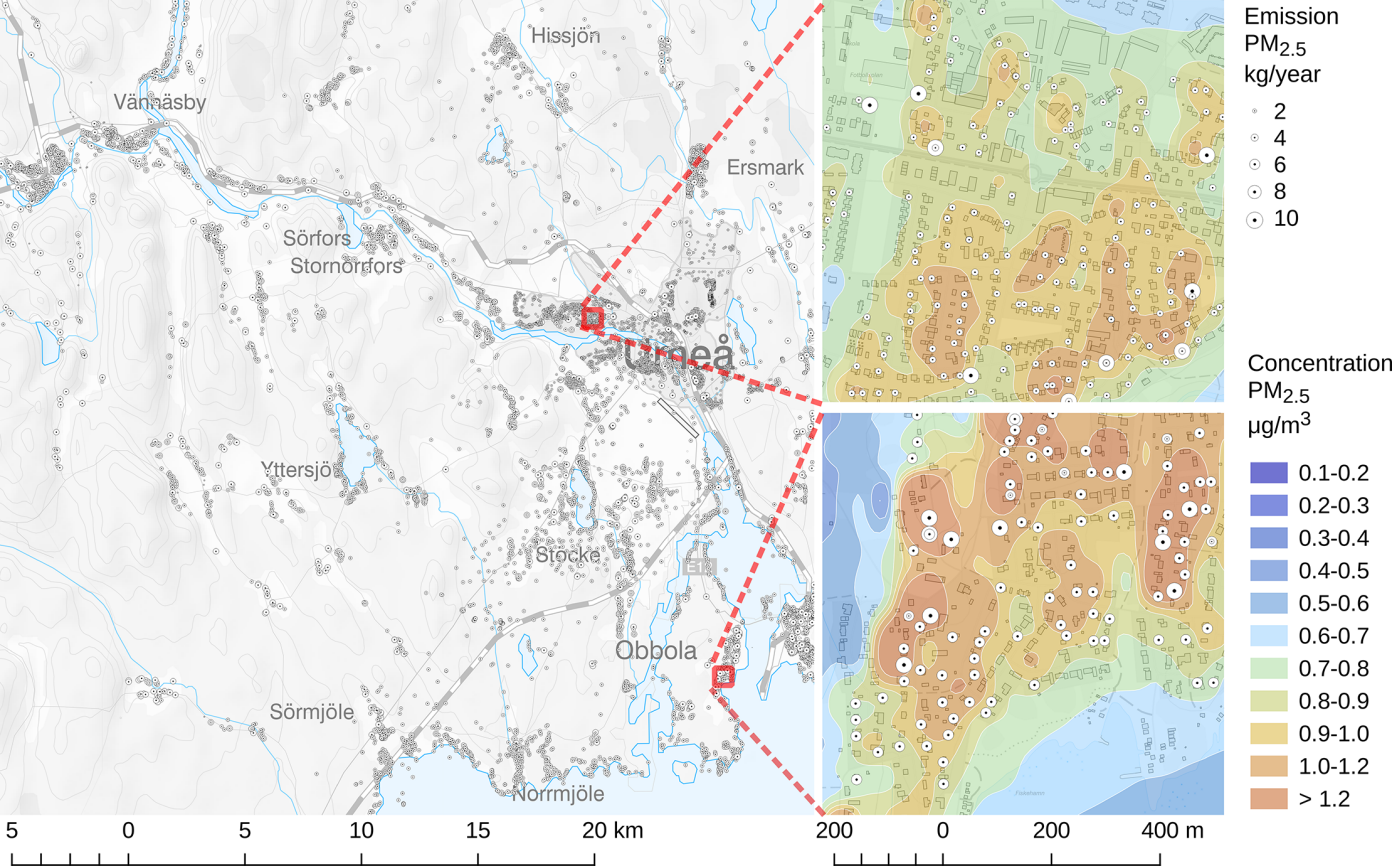
Emissions and modelled concentrations of PM_2.5_ from residential wood burning in two small areas within the study area, by SMHI. Copyright Lantmäteriet.

An example of the detailed data on emissions of PM_2.5_ related to residential wood combustion in the study area is shown in Fig 3.

Descriptive data on all variables, including missing data, are shown in Table 1. The associations between dementia and source-specific PM_2.5_ are shown in Tables 2 and 3, in models adjusted for other risk factors, and including PM_2.5_ from both traffic exhaust and wood burning as separate variables. For example, the hazard ratio (HR) for dementia associated with a 1 μg/m^3^ increase in PM_2.5_ from residential wood burning (independent of PM_2.5_ from traffic exhaust) was 1.55 (95% Confidence Interval (CI): 1.00–2.41, p-value 0.05; Table 2). Participants with a wood stove who lived in an area that was classified as the highest quartile of emissions from residential wood burning had a statistically significantly higher risk of dementia than participants without a stove who lived in the first three quartiles of emissions from residential wood burning (HR = 1.74, CI: 1.10–2.75, p-value 0.02, Table 3). The HR was for men 2.59 (95% CI: 1.34–5.00, p-value 0.01) and for women 1.25 (95% CI: 0.62–2.54, p-value > 0.30), which indicates a difference between men and women, but the p-value for effect modification was not statistically significant (p > 0.30). Particulate matter from traffic exhaust was associated with dementia incidence with hazard ratios of 1.66, (CI: 1.16–2.39, p-value 0.01), and 1.41 (CI: 0.97–2.23, p-value 0.07), in the third and fourth quartiles, respectively (Table 2).

**Table 1:** Distribution of dementia and population characteristics at baseline according to mean PM_2.5_ exposure from vehicle exhaust and residential wood burning [n (%), mean (SD)].

			PM_2.5_ (μg/m^3^)	
			Vehicle exhaust	Residential wood burning
		N(%)	Mean (SD)	Mean (SD)
All		1806(100)	0.18(0.17)	0.77(0.30)
Total dementia Vascular dementia Alzheimer’s disease		302(17)	0.20(0.26)	0.76(0.29)
		111(6)	0.19(0.14)	0.79(0.26)
		191(11)	0.20(0.17)	0.75(0.31)
Sex	Men	773(43)	0.18(0.17)	0.79(0.32)
	Women	1033(57)	0.20(0.17)	0.75(0.28)
Age	55	290(16)	0.17(0.17)	0.79(0.31)
	60	286(16)	0.18(0.19)	0.76(0.33)
	65	286(16)	0.18(0.14)	0.79(0.30)
	70	278(15)	0.18(0.15)	0.72(0.25)
	75	274(15)	0.18(0.13)	0.75(0.32)
	80	266(15)	0.24(0.24)	0.77(0.28)
	85	126(7)	0.22(0.14)	0.81(0.24)
Education	Compulsory	1403(78)	0.19(0.17)	0.76(0.29)
	High school	133(7)	0.18(0.19)	0.80(0.33)
	University	246(14)	0.21(0.19)	0.79(0.26)
	Missing	24(1)	0.20(0.11)	0.90(0.63)
Physical activity	Never	412(23)	0.22(0.21)	0.76(0.25)
	Occasionally	219(12)	0.18(0.15)	0.76(0.32)
	Few times per month	176(10)	0.20(0.16)	0.76(0.28)
	Weekly	588(33)	0.18(0.16)	0.78(0.31)
	Daily	377(21)	0.18(0.17)	0.76(0.31)
	Missing	34(2)	0.19(0.10)	0.69(0.30)
Smoking	Non-smoker	1001(55)	0.18(0.15)	0.77(0.32)
	Smoker	225(12)	0.21(0.22)	0.75(0.23)
	Ex-smoker	580(32)	0.19(0.18)	0.77(0.28)
Alcohol	Yes	1272(70)	0.20(0.18)	0.77(0.30)
	No, never	445(25)	0.16(0.14)	0.76(0.29)
	No, have quit	85(5)	0.16(0.11)	0.75(0.32)
	Missing	4(0)	0.16(0.08)	0.69(0.03)
BMI^1^	Overweight	1180(65)	0.19(0.17)	0.76(0.30)
	Normal or underweight	566(31)	0.19(0.18)	0.78(0.28)
	Missing	60(3)	0.18(0.10)	0.76(0.22)
WHR^2^	>Recommended	697(39)	0.20(0.18)	0.74(0.27)
	≤Recommended	963(53)	0.18(0.17)	0.78(0.31)
	Missing	146(8)	0.18(0.13)	0.78(0.29)
Deceased		589(33)	0.21(0.18)	0.77(0.29)
Type of stove or boiler	none	1325(73)	0.20(0.18)	0.72(0.26)
	wood stove	364(20)	0.17(0.12)	0.87(0.30)
	wood boiler less often used	28(2)	0.07(0.06)	1.10(0.59)
	wood boiler used a lot	38(2)	0.08(0.06)	1.14(0.52)
	pellet or oil	51(3)	0.21(0.17)	0.70(0.25)

^1^ BMI (Body Mass Index). BMI cut-offs between normal weight and overweight were 23.8 for women and 25.0 for men.

^2^ WHR (Waist-hip-ratio). Cut-off of 0.8 for women and 1.0 for men.

**Table 2:** Hazard Ratios (HRs) and 95% Confidence Intervals (CIs) for dementia in association with quartiles of source-specific PM_2.5_ concentration and type of stove or boiler, from cox proportional hazards models.

Residential wood burning PM_2.5_^a^ (μg/m^3^)	n/cases/person-years^a^	HR^a^ (95% CI)	HR^b^ (95% CI)
0.21–0.54	349/66/4437	1	1
0.54–0.72	354/61/4429	0.88(0.63–1.22)	0.88 (0.61–1.25)
0.72–0.91	350/51/4278	0.75 (0.53–1.06)	0.87 (0.60–1.26)
0.91–3.34	349/65/4235	0.97(0.70–1.34)	1.29 (0.91–1.83)
Per 1 μg/m^3^ increase in exposure **Traffic exhaust PM**_**2.5**_^**a**^		1.05(0.70–1.57)	1.55 (1.00–2.41)
0.017–0.086	356/51/4577	1	1
0.086–0.14	356/47/4785	0.95 (0.65–1.38)	1.02 (0.68–1.53)
0.14–0.24	345/76/4140	1.70 (1.21–2.39)	1.66 (1.16–2.39)
0.24–1.81	345/69/3877	1.65 (1.17–2.34)	1.41 (0.97–2.04)
Per 1μg/m^3^ increase in exposure PM_2.5_		1.71(0.94–3.13)	1.14 (0.59–2.23)
**Type of stove or boiler**^**c**^			
none	1028/183/12621		1
wood stove	292/50/3747	0.99(0.75–1.31)	1.17 (0.84–1.62)
wood boiler less often used	21/2/241	0.65(0.21–2.02)	0.54 (0.13-2-23)
wood boiler used a lot	26/4/318	1.11(0.52–2.34)	1.53 (0.55–4.26)
pellet or oil	35/4/450	0.48(0.18–1.30)	0.61 (0.23–1.68)

^a^Crude model and unadjusted estimates

^b^The model includes PM_2.5_ from traffic exhaust, PM_2.5_ from residential wood burning, physical activity, smoking, sex, body mass index, waist-hip-ratio, alcohol and age.

^c^The model includes type of stove or boiler, PM_2.5_ from traffic exhaust, physical activity, smoking, sex, body mass index, waist-hip-ratio, alcohol, age.

**Table 3:** Hazard Ratios (HRs) and 95% Confidence Intervals (CIs) for dementia in association with PM_2.5_ concentration from residential wood burning from cox proportional hazards models.

	n/cases/person-years	HR (95% CI)^a^	HR (95% CI)^b^
quartiles 1–3, without stove	10437/850/149	1	1
quartiles 1–3, with stove	2302/173/27	0.83 (0.57–1.23)	0.97 (0.64–1.47)
quartile 4, without stove	2744/225/40	0.98 (0.70–1.37)	1.19 (0.83–1.71)
quartile 4, with stove	1445/119/23	1.11 (0.73–1.69)	1.74 (1.10–2.75)

^a^Unadjusted estimates

^b^The model includes vehicle exhaust, physical activity, smoking, sex, body mass index), waist-hip-ratio, alcohol and age.

## Discussion

Although previous work has indicated a link between air pollution and dementia incidence, evidence is not yet present to clearly pinpoint which pollutants are the probable causal agents. In particular, the effect of other sources of air pollution than traffic, such as air pollution due to residential wood burning has yet to be explored. Our results provide evidence that not only is particulate matter from traffic exhaust associated with dementia incidence, which supports previous findings on (mainly traffic-related) air pollution, [2–6] but particulate matter from residential wood burning also seem to be associated with dementia incidence in our study area. Previous studies had no source-specific data on air pollution, so this is the first study to separate air pollution from different sources. The high spatial resolution of the air pollution model in the present study resolves intra-city gradients and is expected to increase the accuracy of exposure estimates, especially for sources located in residential areas. The spatial distribution of exposure caused by the two main local sources of PM–road traffic and small scale residential wood burning–was very different, allowing the impact of the different sources to be separated.

Our results suggest a dose-response association between PM_2.5_ from wood smoke and dementia. The quartile point estimates suggest an association in the highest quartile although it is not statistically significant. This may be due to statistical power. For the vehicle exhaust, the point estimates suggest an association in quartile 3 and 4, similar to what we saw in our previous study [2]. There is no evidence for a dose-response association though (linear estimate close to 1). Our findings on vehicle exhaust is basically reproducing the results from our previous study (here we look at PM_2.5_ from vehicle exhaust, which should be highly correlated with NO_x_, which was the exposure indicator in the previous study).

A direct mechanistic pathway between exposure to air pollution and neurodegenerative diseases are plausible, but not yet fully understood. Experimental studies on animal data suggest that PM may reach the brain via circulation or via the olfactory bulb [23–25]. Experimental data further link exposure to air pollution to multiple pathways crucial to dementia pathogenesis [26–31]. A possible, but less established, pathway is that ambient pollutants increase the risk for dementia indirectly, via cardiovascular effects [32–36]. We have previously seen however that cardiovascular diseases did not seem to be the main pathway between air pollution exposure and dementia [2].

It is well established that biomass burning emits health-damaging pollutants, including several carcinogenic compounds [8]. Indoor wood smoke at high doses is a well-established cause of morbidity and mortality but due to lack of exposure data there have been no epidemiological population-based studies on exposure to wood smoke in association with mortality and health. However, PM from wood burning has been estimated to cause a larger or comparable number of annual deaths (depending on location and risk estimate used) than traffic-related air pollution in Sweden [16]. The biomass contribution to air pollution levels in small country towns in Sweden can be comparable to the most polluted street canyons in the capital Stockholm [12]. Biomass burning is increasing in Europe due to climate mitigation policies, but possible health effects of such policy are not known [14, 15]. Our results imply biomass burning as a neglected but possible detrimental contribution to air pollution exposure that merits further attention.

Participants who both lived in areas with the highest quartile of PM_2.5_ from residential wood burning, and had a wood stove in their homes (which should be the group with highest exposure to PM_2.5_ from residential wood burning), had substantially higher dementia incidence than other participants (HR of 1.74, CI: 1.10–2.75). Most of the households in quartiles 1 to 3 of exposure from residential wood burning were located in areas with district (long-distant) heating, and we know from a previous interview study that stoves and boilers in such areas were used to a lesser extent [22]. Furthermore, wood stoves are found indoors and are likely to generate a higher personal exposure than wood boilers. An analysis that was stratified by sex indicated that the risk increase seemed even higher in men (HR = 2.59, CI: 1.34–5.08). A possible explanation for this could be that men have higher exposure than women, for example if males are more prone to handle the boilers and stoves than women. Compared with our previous study, [2] the effect estimates associated with the continuous exposure in the current study may seem high, but that is because the current model only considers local sources, and the local contribution consist of a small fraction of the total concentrations of air pollutants.

The major strength of the study is the new, detailed, exposure models and the assessment of diagnosis. The diagnosis is well-validated and not relying solely on hospital-records of diagnosis. The detailed data on air pollution (PM_2.5_) from residential wood burning has not been used before in studies on air pollution and dementia, and we are not aware of any application of dispersion models with as detailed data on residential wood burning outside Sweden (in Southern Sweden there is one similar model). The model has high spatial resolution and high accuracy, meaning that we could assess exposure on a very detailed level. However, it would have been valuable to have data on individual-level firing habits, since this influences both personal and neighborhood exposure [22]. A possible limitation though is that we used the concentrations of PM_2.5_ for the inclusion year in this study, similar to what we did in our previous study [2]. It would be interesting to also investigate cumulative exposure during follow-up given the nature of dementia, which might increase the risk of exposure misclassification. The probability of changing residential address may increase at symptom onset, when the individual may not be able to reside in their old home anymore, whereas the diagnosis may come years later. Exposure misclassification is always a potential source of bias in studies on exposure to air pollution and health. People are not always at their residences or spend little time outdoors (where air pollution is modelled) and they may be exposed to other sources of air pollution that we cannot account for in our analysis. In this study we were able to adjust for type of stove or boiler (and the estimates did not change much), but there are other sources of air pollution that we could not take into account. Exposure misclassification can in theory create bias both away and towards the null, and it is difficult to completely rule out differential exposure misclassification as an explanation of findings in all studies of air pollution and health. However, the spatial distribution of road traffic and small scale residential wood burning was very different, and differential exposure misclassification most likely would vary between the two sources. Exposure misclassification as an explanation for our findings regarding therefore seem unlikely. Furthermore, residual confounding must always be considered in observational studies. We considered several potential confounding factors, but only adjustment for age had any substantial influence on the association between the exposures and dementia incidence. Factors related to socioeconomic status, such as education and smoking, had no influence on the observed associations. In all studies of traffic-related air pollution as a risk factor for incident disease, residual confounding from unknown geographically varying urban factors is always difficult to rule out. In the present study, traffic noise could be such a factor. However, Power and colleagues emphasize that their review suggests residual confounding is unlikely to account for the consistently positive results between exposure to air pollution and dementia-related outcomes that have so far observed [7]. The results of our study support that statement, as the geographical gradients in air pollution from residential wood burning are substantially different from traffic-related gradients (Fig 2), and we observe associations with particles from both residential wood burning and vehicle exhaust. For residual confounding from a geographically varying factor to explain our results, there would thus have to be two different such factors, which seems unlikely. Furthermore, in their review Power and colleagues identify no major limitations with our previous study, which was identical to the present study except the new more detailed exposure data [7].

The results of the present study cannot be generalized to those invited to the Betula study who declined to participate, and participation tends to depend on socio-economic status, age and sex. Air pollution is a complex mixture that varies in time and space, and the generalizability of the results from studies on air pollution and health are always difficult to assess. The source-specific air pollution estimates in the present study should increase the generalizability of the results compared to studies where exposure is less specific however. We have earlier ruled out non-informative censoring to be a plausible explanation for the association between traffic-related air pollution and incident dementia [2]. The levels of particulate air pollution from residential wood burning seem similar in the deceased as in the entire study, (Table 1) why differential mortality due to exposure does not explain our findings.

In conclusion, if the associations we observed are causal, then air pollution from residential wood burning, and air pollution from traffic, might be independent important risk factors for cardiovascular dementia and Alzheimer’s disease. Given that this is the first study on exposure to residential wood burning and dementia incidence, the results should be corroborated by others.
